# TRAJECTORIES AND CORRELATES OF POST-LOSS DEPRESSIVE SYMPTOMS IN FORMER DEMENTIA CAREGIVERS

**DOI:** 10.1093/geroni/igz038.410

**Published:** 2019-11-08

**Authors:** Kristin L Corey, Karen Hirschman, Lauren T Starr, Salimah H Meghani

**Affiliations:** 1 NewCourtland Center for Transitions and Health, University of Pennsylvania School of Nursing, Philadelphia, Pennsylvania, United States; 2 University of Pennsylvania, Philadelphia, Pennsylvania, United States

## Abstract

Approximately 30-40% of family caregivers of relatives living with dementia report depression, compared to 9.5% of the general adult population. Studies suggest that depressive symptoms persist for many years following the care recipient’s death, despite resolution of caregiving responsibilities. However, long-term patterns of post-loss depressive symptoms remain poorly understood. The aim of this integrative review was to examine longitudinal trajectories and correlates of depressive symptoms in dementia caregivers following the care recipient’s death. A literature search was conducted using CINAHL, MEDLINE, and PsycINFO databases. Studies met the eligibility criteria if they were peer-reviewed, primary sources and reported research exploring correlates and/or longitudinal patterns of post-loss depressive symptoms in dementia caregivers. Data quality was evaluated using the widely-used quality appraisal tool developed by Hawker and colleagues (2002). Data were extracted, displayed in matrix format, and synthesized into themes. Fourteen studies met the eligibility criteria and were rated as high quality. Overall, depressive symptom severity trended down during the first year post-loss but did not reduce to levels reported by non-caregiving controls. Symptom trajectories varied among unique caregiver subgroups and included persistent-distressing symptoms, persistent-mild symptoms, and asymptomatic. Greater severity of post-loss depressive symptoms was associated with female gender, lower income, less social support, pre-loss depression, neurotic and optimistic personality traits, and avoidant coping style. The findings indicate that many caregivers could benefit from mental health screening and psychosocial support during the first year post-loss and underscore the need for longitudinal studies that explore depressive symptom trajectories beyond the first 1-2 years post-loss.

